# Complications of percutaneously placed uncovered metallic biliary stents for malignant obstruction: a systematic review

**DOI:** 10.3389/fradi.2025.1639323

**Published:** 2025-08-05

**Authors:** Jonathan Bock, Christopher J. Reisenauer, Michael C. Jundt, Matthew R. Augustine, Richard G. Frimpong, Edwin A. Takahashi

**Affiliations:** ^1^ Mayo Clinic Alix School of Medicine, Mayo Clinic, Rochester, MN, United States; ^2^ Department of Radiology, Division of Vascular and Interventional Radiology, Mayo Clinic, Rochester, MN, United States

**Keywords:** bile duct, biliary stent, malignant biliary obstruction, complications, outcomes

## Abstract

**Background:**

The aim of this systematic review was to determine the patency and complications related to percutaneous metallic biliary stent placement for malignant biliary obstruction in the current literature.

**Methods:**

This review was performed using the Preferred Reporting Items of Systematic Reviews and Meta-Analyses guidelines. EMBASE and PubMed were queried yielding 891 articles, 18 of which were included in the final analysis. The Newcastle-Ottawa Quality Assessment Scale was used to appraise article quality. Patient demographics, technical success rate, and procedure outcomes were recorded. Complications were classified as “major” if they resulted in blood transfusion or additional invasive procedures or were reported as such in the literature. Complications that did not meet these criteria were classified as “minor”.

**Results:**

A total of 1,453 patients (677 female; weighted age 66.8 years) underwent biliary stent placement. The weighted technical success rate was 97.7%. The incidence of stent occlusion was 13.5% with 6.6% of patients requiring further intervention to maintain patency. There were 277 (19.1%) complications, of which 87 were classified as major. The most common complications were pancreatitis (93, 6.4%), cholangitis (69, 4.8%), and bleeding (64, 4.4%). In cases of bleeding, 4.7% of patients needed a blood transfusion and 15.6% required a procedure to treat bleeding. There were 6 (0.4%) procedure-related deaths.

**Conclusion:**

In conclusion, percutaneous metallic stent placement for malignant biliary obstruction has a high technical success rate and relatively low rate of occlusion. Although nearly one in five procedures resulted in a complication, most cases were minor.

## Introduction

Unresectable malignant biliary obstruction is a common consequence of pancreatic adenocarcinoma, cholangiocarcinoma, hepatocellular carcinoma and gallbladder carcinoma (1). This condition may lead to jaundice, pruritus, and cholangitis, significantly increasing morbidity and compounding the burdens associated with cancer progression. Tumor invasion and metastatic spread frequently preclude curative resection and limit treatment options for patients with biliary obstruction.

Stent placement is a widely accepted treatment for unresectable malignant biliary obstruction, offering symptom palliation and improved quality of life (2, 3). The percutaneous approach to stent placement has demonstrated safety and efficacy. Bare metal stents may be preferred over covered stents due to operator experience, cost, and potentially lower rates of stent migration (4). However, several complications can occur during stent placement including hemorrhage, bile leakage, and pancreatitis (5). Additionally, these stents can occlude and necessitate secondary interventions for biliary diversion or stent recanalization.

The purpose of this study was to evaluate the outcomes of percutaneously placed uncovered metallic biliary stents for the treatment of malignant biliary obstruction in the current literature with particular focus on stent patency and complications.

## Methods

### Search strategy

**Figure 1:**
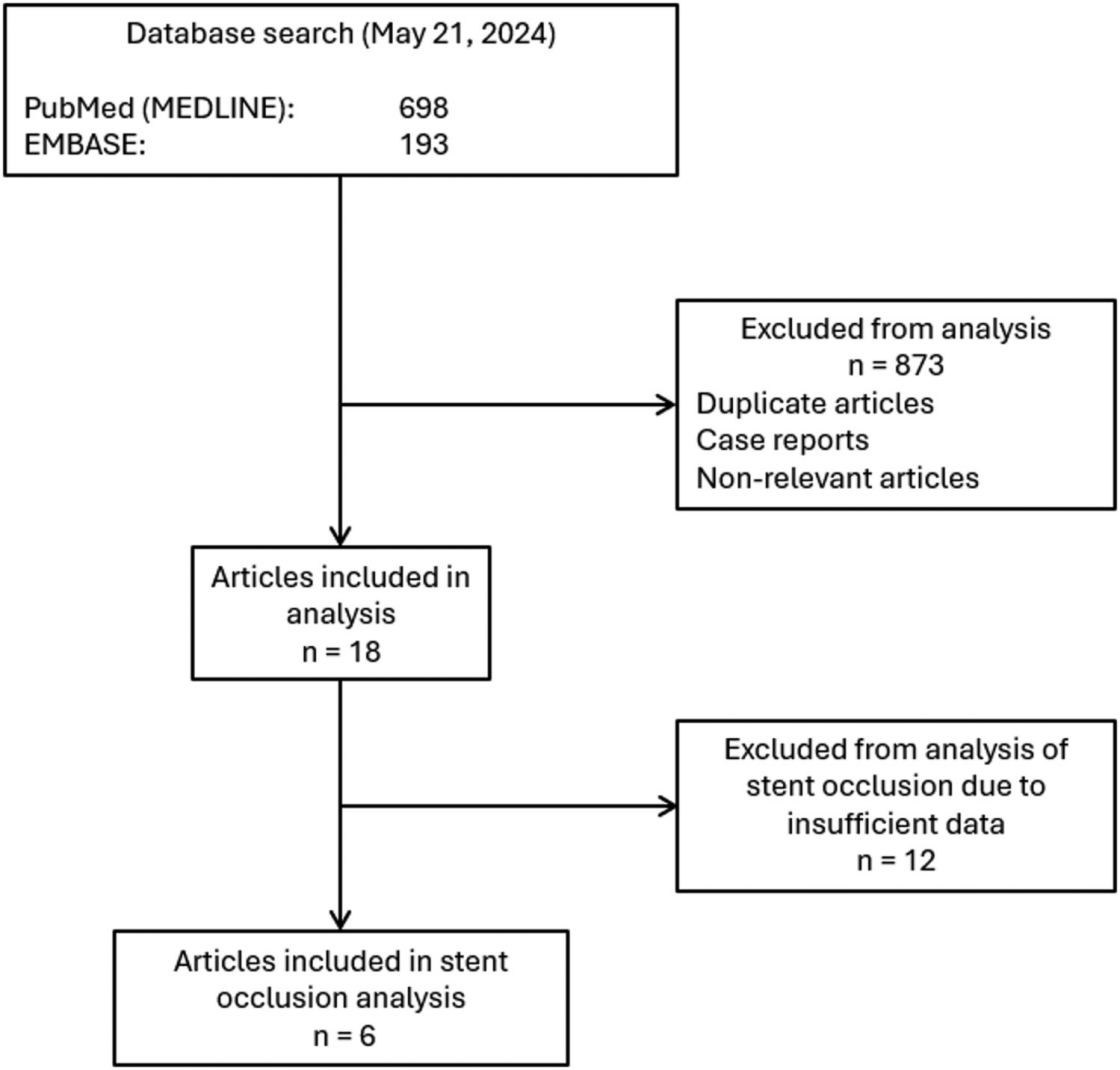
Flow diagram of search strategy.

This systematic review was conducted following the Preferred Reporting Items of Systematic Reviews and Meta-Analyses (PRISMA) guidelines (6). Institutional review board (IRB) approval was precluded by the study design. Articles published on or before May 2024 were identified using the terms: “percutaneous” AND [biliary OR “bile duct” (MeSH)] AND [stent or “stents” (MeSH)], AND “placement”. This query yielded 193 articles in EMBASE and 698 articles in PubMed. A total of 891 article abstracts were reviewed. Duplicate articles, case reports, and nonrelevant articles were excluded. The final analysis included 18 articles published from 2002 to 2022 (7–24). A summary of the search strategy is shown in Figure 1.

### Quality assessment

The Newcastle-Ottawa Scale (NOS) for cohort studies was used to appraise article quality (25). Two independent reviewers assessed each article to evaluate the selection methods, comparability, and outcome robustness of the study. These three metrics were used to calculate a numeric total that correlates with the level of evidence (good, fair, or poor). Discrepancies between the reviewers were resolved by consensus.

### Data extraction and outcome measures

Raw data extraction was performed on articles that met the inclusion criteria. Patient demographics, technical success rate, procedure outcomes regarding patency rates and number of re-interventions, and complications were recorded. Weighted means were performed where possible. Complications recorded included bleeding, pancreatitis, cholecystitis, cholangitis, other infection, peritonitis/bile leak, pneumonia, and pneumothorax. Complications were classified as “major” if they resulted in blood transfusion, required additional invasive procedures (e.g., arterial embolization), or were reported as such by the authors of a given study. Complications that did not meet these criteria were classified as “minor” in accordance with the Society of Interventional Radiology (SIR) guidelines (26). The average duration of stent patency was collected when available (6 of 18 studies), providing an additional metric for determining the efficacy of stent placement.

## Results

According to NOS criteria, 16 of the 18 articles were rated as “poor” due to lack of a comparison group within the study. Two other articles that compared covered and uncovered stents with matched cohorts based on age and sex received a “fair” quality rating (15, 19).

A total of 1,453 patients (677 female) underwent biliary stent placement. The weighted mean patient age was 66.8 years. Two studies were excluded from age analysis due to incomplete demographic data (27, 28). Table 1 summarizes patient characteristics. The weighted technical success rate of percutaneous metallic biliary stent placement was 97.7%. Hilar lesions were treated in 845 patients and ampullary lesions treated in 451 patients. Lesion location could not be determined from 3 studies (19, 20, 24).

**Table 1:** Summary of patient demographics.

Characteristic	
Number of patients	1,453
Weighted mean age, years	66.8 years
Male: female	776:677
Malignancy, *n*	
Cholangiocarcinoma	549
Gallbladder carcinoma	160
Pancreatic	296
Hepatocellular carcinoma	121
Duodenal/ampullary	16
Not specified/metastatic	311

### Patency outcomes

The mean stent primary patency duration ranged from 114.7 ± 15.1 days to 413 ± 63.0 days (7, 8, 13–15, 21). In the 6 articles that contained data on stent occlusion, the weighted mean time to occlusion was 83.9 days, ranging from raw means of 50.2 days to 189 days (8, 13, 14, 16, 19, 21). In total, 196 (13.5%) patients developed stent occlusion. Furthermore, 6.6% of patients required another procedure to recanalize their stent. Post-stent biliary drains were placed in 3.9% of cases for biliary diversion.

### Safety outcomes

There were 277 complications (19.1% of patients), of which 87 (6.0%) were classified as major. The most common complications were pancreatitis (93, 6.4%), cholangitis (69, 4.8%), and bleeding (64, 4.4%). Additional complications included other infection, peritonitis/bile leak, pneumonia, cholecystitis, and pneumothorax (27 [1.9%], 9 [0.6%], 9 [0.6%], 4 [0.3%], and 2 [0.1%], respectively).

Among the 64 cases of bleeding complication, 3 (4.7%) patients needed a blood transfusion and 10 (15.6%) patients underwent a procedure such as transarterial embolization to control the bleeding. There were 6 procedure-related deaths (0.4%) associated with stent placement. Patency data and complication rates from each study are summarized in Table 2.

**Table 2:** Outcomes of noncovered biliary stent placement for malignant obstruction.

Study, year (ref#)	No. of patients	Technical Success (%)	Stent occlusion events *n* (%)	Mean time to stent occlusion days (SD* or range^†^)	Complications *n* (%)	
					Minor	Major
Ahn et al, 2,012 (7)	26	92	8 (30.8)		0 (0.0)	2 (7.7)
Brountzos et al, 2006 (8)	76	100	9 (11.8)	61 (11–135)^†^	6 (7.9)	11 (14.5)
Dhondt et al, 2020 (9)	78	100	20 (25.6)		19 (24.4)	7 (9.0)
Fu et al, 2019 (10)	72	83	8 (11.1)		6 (8.3)	0 (0.0)
Fucilli et al, 2019 (11)	45	100	0 (0.0)		1 (22.2)	1 (2.2)
Gwon et al, 2011 (12)	41	100	8 (19.5)		12 (29.3)	0 (0.0)
Han et al, 2006 (13)	17	94	2 (11.8)	189 (111.5)*	4 (23.5)	3 (17.6)
Krokidis et al, 2010 (14)	40	100	12 (30.0)	83 (20.1)*	4 (10.0)	0 (0.0)
Lee et al, 2014 (15)	20	100	4 (20.0)		0 (0.0)	0 (0.0)
Li et al, 2016 (16)	92	100	36 (39.1)	91 (21–343)^†^	0 (0.0)	20 (21.7)
Mao et al, 2017 (18)	41	100	15 (36.6)		23 (56.1)	0 (0.0)
Mao et al, 2021 (17)	80	98	18 (22.5)		8 (10.0)	1 (1.3)
Onishi et al, 2020 (19)	30	100	6 (20.0)	50.2 (30.1)*	5 (16.7)	0 (0.0)
Pinol et al, 2002 (20)	28	75	12 (42.9)		0 (0.0)	16 (57.1)
Pranculis et al, 2017 (21)	222	96	16 (7.2)	81 (4–264)^†^	10 (4.5)	21 (10.0)
Xu et al, 2022 (22)	425	100	0 (0.0)		40 (9.4)	1 (0.2)
Zhang et al, 2019 (23)	21	100	9 (42.9)		1 (4.8)	4 (19.0)
Zurstrassen et al, 2017 (24)	99	100	13 (13.1)		51 (51.5)	0 (0.0)

## Discussion

Percutaneous metallic biliary stent placement is a common palliative therapy for malignant biliary obstruction. However, much the existing literature on this procedure is limited to small retrospective studies. This systematic review consolidates the current data to better characterize stent placement outcomes. The analysis revealed that percutaneous uncovered metallic biliary stent placement has a high technical success rate, low stent occlusion rate, and a procedure-related mortality rate of less than 1%. While most complications were minor, the overall complication rate of approximately 19.1% is noteworthy.

The technical success rate of percutaneous biliary stent placement surpasses that of the endoscopic approach. A study in 2009 by Paik et al. comparing these methods in patients with advanced hilar cholangiocarcinoma reported a technical success rate of 92.7% in the percutaneous group vs. 77.3% in the endoscopic group (29). Similarly, a randomized clinical trial by Pinol et al. reported a technical success rate of 75% for percutaneous placement compared to 58% for endoscopic placement. This study also found higher therapeutic success rates in the percutaneous group (71% vs. 61%) (20). Additionally, percutaneous approaches are often preferred when initial endoscopic attempts are unsuccessful (30, 31).

The rate of stent occlusion among the analyzed studies was low at 13.5% with a weighted mean time to occlusion of 83.9 days. Uncovered stents were the focus of this review. The literature presents mixed findings regarding potential superior patency of covered stents. Some studies report no significant difference in patency or complication rates between covered and uncovered stents (4, 15, 19). For example, a randomized multicenter trial of 400 patients with malignant biliary distal biliary obstruction found no significant differences between covered and uncovered metallic stents placed endoscopically (*p* = 0.30) (32). Conversely, a randomized trial of 80 patients comparing percutaneously placed stents for pancreatic cancer reported a mean patency of 166 days vs. 234 days for uncovered and covered stents (*p* < 0.01), respectively (14). Overall, this systematic review highlights that palliative percutaneous biliary stenting for unresectable malignancies helped the majority of patients achieve internal bile drainage without needing secondary procedures to maintain patency or adding morbidity to end of life care.

The total complication rate among the articles reviewed was 19.1% with nearly one third of these events considered to be “major”. The most common complication was pancreatitis, accounting for 33.6% of the 277 complications, though the severity of these cases remains unclear due to inconsistent reporting across studies. While limited literature directly compares percutaneous and endoscopic biliary metallic stent placement, available data suggest endoscopic techniques have similar or higher complication rates. For example, a retrospective study of 4,623 patients with endoscopically placed biliary stents for malignant biliary obstruction reported an adverse event rate of 15.7%, with pancreatitis being the most frequent complication (4.7%) (33). Ho et al. reported an 18% complication rate with endoscopically placed partially covered biliary metal stents (34). A 2023 study by Paik et al. found a combined adverse event rate of 32% for metallic and plastic stents that were placed endoscopically for malignant biliary obstruction (35). Additionally, in a prospective study on malignant hilar obstruction, De Palma et al. reported early complications in 8.2% of patients and late complications in 22.9% of patients with endoscopically placed metallic stents (36). Further comparative studies are needed to clarify the outcome differences between percutaneous and endoscopic metallic stent placement for malignant obstruction.

This study has several limitations primarily due to its retrospective nature and the overall quality of the included articles, most of which received a “poor” rating based on NOS criteria. The heterogeneity of the data precluded meta-analysis, and there was inconsistent reporting of complication severity, repeat interventions, and stent patency duration. Patient follow-up was not standardized. Additionally, this systematic review focused on uncovered stents due to their widespread use and to avoid confounding factors related to stent design, which may obscure conclusions on patency and complications.

Percutaneous uncovered metallic stent placement for malignant biliary obstruction has a high technical success rate and very low procedure-related mortality. The need for secondary interventions to maintain stent patency or achieve biliary diversion was low. However, while most complications were minor, the overall complication rate was 19.1%, underscoring the importance of careful patient selection to optimize palliative care for those with unresectable biliary malignancies.

## Data Availability

The original contributions presented in the study are included in the article/Supplementary Material, further inquiries can be directed to the corresponding author.

## References

[1] Lorenz JM. Management of malignant biliary obstruction. Semin Intervent Radiol. (2016) 33(4):259–67. 10.1055/s-0036-1592330 27904244 PMC5088103

[2] Abraham NS Barkun JS Barkun AN. Palliation of malignant biliary obstruction: a prospective trial examining impact on quality of life. Gastrointest Endosc. (2002) 56(6):835–41. 10.1067/mge.2002.129868 12447294

[3] Lee TH Moon JH Park SH. Biliary stenting for hilar malignant biliary obstruction. Dig Endosc. (2020) 32(2):275–86. 10.1111/den.13549 31578770

[4] Chen MY Lin JW Zhu HP Zhang B Jiang GY Yan PJ Covered stents versus uncovered stents for unresectable malignant biliary strictures: a meta-analysis. Biomed Res Int. (2016) 2016:6408067. 10.1155/2016/6408067 27051667 PMC4802019

[5] Song J Deng J Wen F. Risk factors associated with acute pancreatitis after percutaneous biliary intervention: we do not know nearly enough. Gastroenterol Res Pract. (2023) 2023:9563074. 10.1155/2023/9563074 36644482 PMC9839406

[6] Moher D Liberati A Tetzlaff J Altman DG. Preferred reporting items for systematic reviews and meta-analyses: the PRISMA statement. Br Med J. (2009) 339:b2535. 10.1136/bmj.b2535 19622551 PMC2714657

[7] Ahn SJ Bae JI Han TS Won JH Kim JD Kwack KS Percutaneous biliary drainage using open cell stents for malignant biliary hilar obstruction. Korean J Radiol. (2012) 13(6):795–802. 10.3348/kjr.2012.13.6.795 23118579 PMC3484301

[8] Brountzos EN Ptochis N Panagiotou I Malagari K Tzavara C Kelekis D. A survival analysis of patients with malignant biliary strictures treated by percutaneous metallic stenting. Cardiovasc Intervent Radiol. (2007) 30(1):66–73. 10.1007/s00270-005-0379-3 17031733

[9] Dhondt E Vanlangenhove P De Man M Huyck L Defreyne L. No advantage of expanded polytetrafluoroethylene and fluorinated ethylene propylene-covered stents over uncovered nitinol stents for percutaneous palliation of malignant infrahilar biliary obstruction: results of a single-center prospective randomized trial. J Vasc Interv Radiol. (2020) 31(1):82–92. 10.1016/j.jvir.2019.07.013 31627908

[10] Fu YF Zhou WJ Shi YB Cao W Cao C. Percutaneous stenting for malignant hilar biliary obstruction: a randomized controlled trial of unilateral versus bilateral stenting. Abdom Radiol (NY). (2019) 44(8):2900–8. 10.1007/s00261-019-02010-6 30968181

[11] Fucilli F Licinio R Lorusso D Giorgio P Caruso ML. One stage percutaneous transhepatic biliary stenting for malignant jaundice: a safe, quick and economical option of treatment. Eur Rev Med Pharmacol Sci. (2019) 23(17):7684–93. 10.26355/eurrev_201909_18892 31539161

[12] Gwon DI Ko GY Sung KB Yoon HK Shin JH Hyoung Kim J Percutaneous biliary metallic stent placement in patients with unilobar portal vein occlusion caused by advanced hilar malignancy: outcome of unilateral versus bilateral stenting. AJR Am J Roentgenol. (2011) 197(4):795–801. 10.2214/AJR.11.6424 21940566

[13] Han YH Kim MY Kim SY Kim YH Hwang YJ Seo JW Percutaneous insertion of zilver stent in malignant biliary obstruction. Abdom Imaging. (2006) 31(4):433–8. 10.1007/s00261-005-8017-8 16465567

[14] Krokidis M Fanelli F Orgera G Tsetis D Mouzas I Bezzi M Percutaneous palliation of pancreatic head cancer: randomized comparison of ePTFE/FEP-covered versus uncovered nitinol biliary stents. Cardiovasc Intervent Radiol. (2011) 34(2):352–61. 10.1007/s00270-010-9880-4 20467870

[15] Lee SJ Kim MD Lee MS Kim IJ Park SI Won JY Comparison of the efficacy of covered versus uncovered metallic stents in treating inoperable malignant common bile duct obstruction: a randomized trial. J Vasc Interv Radiol. (2014) 25(12):1912–20. 10.1016/j.jvir.2014.05.021 25085230

[16] Li M Li K Qi X Wu W Zheng L He C Percutaneous transhepatic biliary stent implantation for obstructive jaundice of perihilar cholangiocarcinoma: a prospective study on predictors of stent patency and survival in 92 patients. J Vasc Interv Radiol. (2016) 27(7):1047–55.e2. 10.1016/j.jvir.2016.02.035 27241392

[17] Mao X Wen F Liang H Sun W Lu Z. A preliminary single-center investigation of percutaneous biliary stenting in malignant hilar biliary obstruction: what impacts the clinical success and the long-term outcomes? Support Care Cancer. (2021) 29(11):6781–92. 10.1007/s00520-021-06271-0 33990879

[18] Mao XN Lu ZM Wen F Liang HY Guo QY. Bare-metal stents across the vater’s ampulla is a safe method for patients with lower bile duct obstruction. Medicine (Baltimore). (2017) 96(45):e7475. 10.1097/MD.0000000000007475 29137005 PMC5690698

[19] Onishi Y Yoshioka T Arai Y Inaba Y Saito H Aramaki T Randomized controlled study to compare uncovered stent versus covered stent as percutaneous endoprosthesis for malignant biliary obstruction (JIVROSG-0207). Am J Clin Oncol. (2020) 43(11):784–7. 10.1097/COC.0000000000000750 32826390

[20] Pinol V Castells A Bordas JM Real MI Llach J Montana X Percutaneous self-expanding metal stents versus endoscopic polyethylene endoprostheses for treating malignant biliary obstruction: randomized clinical trial. Radiology. (2002) 225(1):27–34. 10.1148/radiol.2243011517 12354980

[21] Pranculis A Kievisas M Kievisiene L Vaicius A Vanagas T Kaupas RS Percutaneous transhepatic biliary stenting with uncovered self-expandable metallic stents in patients with malignant biliary obstruction - efficacy and survival analysis. Pol J Radiol. (2017) 82:431–40. 10.12659/PJR.901785 29662569 PMC5894070

[22] Xu C Xu GX Liu S Shi HB Zhou WZ. Acute pancreatitis after percutaneous metallic stent insertion for malignant biliary obstruction: a retrospective 2-center study. Turk J Gastroenterol. (2023) 34(9):961–7. 10.5152/tjg.2023.22442 37565796 PMC10544230

[23] Zhang JX Liu J Wang B Liu S Zu QQ Shi HB. Retrospective comparison of different percutaneous approaches to manage occluded primary uncovered self-expandable metal stents in patients with unresectable malignant hilar biliary obstruction. Scand J Gastroenterol. (2019) 54(11):1397–402. 10.1080/00365521.2019.1683602 31656114

[24] Zurstrassen CE Bitencourt AGV Guimaraes MD Cavalcante A Tyng CJ Amoedo MK Percutaneous stent placement for the treatment of malignant biliary obstruction: nitinol versus elgiloy stents. Radiol Bras. (2017) 50(2):97–102. 10.1590/0100-3984.2015.0183 28428652 PMC5396999

[25] Wells G Shea B O'Connell D Robertson J Peterson J Welch V The Newcastle-Ottawa Scale (NOS) for Assessing the Quality of Nonrandomised Studies in Meta-analyses. Ottawa, ON: University of Ottawa (2013).

[26] Sacks D McClenny TE Cardella JF Lewis CA. Society of interventional radiology clinical practice guidelines. J Vasc Interv Radiol. (2003) 14(9 Pt 2):S199–202. 10.1097/01.rvi.0000094584.83406.3e 14514818

[27] Al Nakshabandi A Ali FS Albustami I Hwang H Qiao W Johnston NC Biliary drainage in hilar and perihilar cholangiocarcinoma: 25-year experience at a tertiary cancer center. Gastrointest Endosc. (2024) 99(6):938–49.e15. 10.1016/j.gie.2023.12.006 38092128

[28] Pappas P Leonardou P Kurkuni A Alexopoulos T Tzortzis G. Percutaneous insertion of metallic endoprostheses in the biliary tree in 66 patients: relief of the obstruction. Abdom Imaging. (2003) 28(5):678–83. 10.1007/s00261-003-0004-3 14628875

[29] Paik WH Park YS Hwang JH Lee SH Yoon CJ Kang SG Palliative treatment with self-expandable metallic stents in patients with advanced type III or IV hilar cholangiocarcinoma: a percutaneous versus endoscopic approach. Gastrointest Endosc. (2009) 69(1):55–62. 10.1016/j.gie.2008.04.005 18657806

[30] Levy MJ Baron TH Gostout CJ Petersen BT Farnell MB. Palliation of malignant extrahepatic biliary obstruction with plastic versus expandable metal stents: an evidence-based approach. Clin Gastroenterol Hepatol. (2004) 2(4):273–85. 10.1016/s1542-3565(04)00055-2 15067620

[31] Jang SI Hwang JH Lee KH Yu JS Kim HW Yoon CJ Percutaneous biliary approach as a successful rescue procedure after failed endoscopic therapy for drainage in advanced hilar tumors. J Gastroen Hepatol. (2017) 32(4):932–8. 10.1111/jgh.13602 27665310

[32] Kullman E Frozanpor F Soderlund C Linder S Sandstrom P Lindhoff-Larsson A Covered versus uncovered self-expandable nitinol stents in the palliative treatment of malignant distal biliary obstruction: results from a randomized, multicenter study. Gastrointest Endosc. (2010) 72(5):915–23. 10.1016/j.gie.2010.07.036 21034892

[33] Lubbe J Sandblom G Arnelo U Jonas E Enochsson L. Endoscopic stenting for malignant biliary obstruction: results of a nationwide experience. Clin Endosc. (2021) 54(5):713–21. 10.5946/ce.2021.016 34058800 PMC8505180

[34] Ho H Mahajan A Gosain S Jain A Brock A Rehan ME Management of complications associated with partially covered biliary metal stents. Dig Dis Sci. (2010) 55(2):516–22. 10.1007/s10620-009-0756-x 19267200

[35] Paik WH Jung MK Kim DU Song TJ Yang MJ Choi YH Side-by-side placement of fully covered metal stents versus conventional 7F plastic stents in malignant hilar biliary obstruction: prospective randomized controlled trial. Dig Endosc. (2024) 36(4):473–80. 10.1111/den.14669 37612129

[36] De Palma GD Pezzullo A Rega M Persico M Patrone F Mastantuono L Unilateral placement of metallic stents for malignant hilar obstruction: a prospective study. Gastrointest Endosc. (2003) 58(1):50–3. 10.1067/mge.2003.310 12838220

